# Cell-type-specific synaptic modulation of mAChR on SST and PV interneurons

**DOI:** 10.3389/fpsyt.2022.1070478

**Published:** 2023-01-12

**Authors:** Huanxin Chen, Ting He, Meiyi Li, Chunlian Wang, Chen Guo, Wei Wang, Baocong Yu, Jintao Huang, Lijun Cui, Ping Guo, Yonggui Yuan, Tao Tan

**Affiliations:** ^1^Huzhou Third Municipal Hospital, The Affiliated Hospital of Huzhou University, Huzhou, Zhejiang, China; ^2^Key Laboratory of Cognition and Personality of the Ministry of Education, School of Psychology, Southwest University, Chongqing, China; ^3^Oujiang Laboratory (Zhejiang Lab for Regenerative Medicine, Vision and Brain Health), Key Laboratory of Alzheimer’s Disease of Zhejiang Province, Institute of Aging, Wenzhou Medical University, Wenzhou, Zhejiang, China; ^4^Department of Neuroscience, Baylor College of Medicine, Houston, TX, United States; ^5^Ningxia Key Laboratory of Craniocerebral Disease, Ningxia Medical University, Yinchuan, China; ^6^Department of Psychosomatic Medicine, Zhongda Hospital, Southeast University, Nanjing, China

**Keywords:** muscarinic acetylcholine receptors (mAChRs), scopolamine, GABAergic interneurons, SST, PV, excitatory and inhibitory synaptic transmission, anterior cingulate cortex

## Abstract

The muscarinic acetylcholine receptor (mAChR) antagonist, scopolamine, has been shown to have a rapid antidepressant effect. And it is believed that GABAergic interneurons play a crucial role in this action. Therefore, characterizing the modulation effects of mAChR on GABAergic interneurons is crucial for understanding the mechanisms underlying scopolamine’s antidepressant effects. In this study, we examined the effect of mAChR activation on the excitatory synaptic transmissions in two major subtypes of GABAergic interneurons, somatostatin (SST)- and parvalbumin (PV)-expressing interneurons, in the anterior cingulate cortex (ACC). We found that muscarine, a mAChR agonist, non-specifically facilitated the frequency of spontaneous excitatory postsynaptic currents (sEPSCs) in both SST and PV interneurons. Scopolamine completely blocked the effects of muscarine, as demonstrated by recovery of sESPCs and mEPSCs in these two types of interneurons. Additionally, individual application of scopolamine did not affect the EPSCs of these interneurons. In inhibitory transmission, we further observed that muscarine suppressed the frequency of both spontaneous and miniature inhibitory postsynaptic currents (sIPSCs and mIPSCs) in SST interneurons, but not PV interneurons. Interestingly, scopolamine directly enhanced the frequency of both sIPSCs and mIPSCs mainly in SST interneurons, but not PV interneurons. Overall, our results indicate that mAChR modulates excitatory and inhibitory synaptic transmission to SST and PV interneurons within the ACC in a cell-type-specific manner, which may contribute to its role in the antidepressant effects of scopolamine.

## 1. Introduction

Muscarinic acetylcholine receptors (mAChRs) play crucial roles in learning, memory, cognition, and psychiatric disorders such as schizophrenia and depression (1–3). mAChRs have thus become an appealing target for developing therapeutic drugs for neurological and psychiatric diseases (4–6). Clinical studies discovered that scopolamine, a mAChR antagonist, exerts rapid and sustained antidepressant effects in patients with depression (7–9). The action was also demonstrated in various animal models of depression (10). It is widely believed that scopolamine initially enhances the glutamatergic activity, promoting the release of brain-derived neurotrophic factors and activating the mechanistic target of the rapamycin signaling pathway (mTOR) leading to synaptogenesis (11–14). However, the mechanisms underlying scopolamine’s enhancement of glutamatergic activity remain elusive.

GABAergic interneurons have been implicated in the pathophysiology of depression and the antidepressant processes (15–17). Previous research has indicated that somatostatin (SST)- and parvalbumin (PV)-expressing interneurons, two major subtypes of interneurons, play a crucial role in antidepressant effects (18, 19). It has been reported that inhibition of PV and SST interneurons elicits a rapid antidepressant action (20). Moreover, the knockdown of M1 mAChRs in SST interneurons in the prefrontal cortex (PFC) prevents scopolamine’s antidepressant action (21). These findings highlight the important role of interneurons in scopolamine’s rapid antidepressant action. However, the mechanism by which scopolamine affects synaptic activity in interneurons remains to be determined.

The anterior cingulate cortex (ACC) is a key PFC region associated with depression (3, 22). A variety of changes in the ACC has been demonstrated in depressed patients, including reduced volume and reduced GABA and glutamate concentration (23–27). A deficit of GABAergic function is suggested in the ACC in patients with depression (28–31). Thus, GABAergic neurons in the ACC possibly participate in scopolamine’s rapid antidepressant action. To explore this possibility, it is crucial to know the regulation of mAChRs and scopolamine on synaptic activities to interneurons. In the present study, we used SST-cre/Ai9 and PV-cre/Ai9 mice to examine the effect of mAChRs and scopolamine on synaptic activities to SST and PV interneurons in the ACC. Our results would provide insight into the initial mechanisms underlying scopolamine’s antidepressant action.

## 2. Materials and methods

### 2.1. Animals

SST-Cre, PV-Cre, and Ai9-red fluorescent protein (RFP) mice were provided by the key laboratory of developmental genes and human diseases, School of Medicine, Southeast University, Nanjing, China. C57BL/6J mice were purchased from Tengxing Biotechnology Company (Chongqing, China). The SST-Cre or PV-Cre mice were bred with Ai9-RFP mice to obtain transgenic mice expressing RFP in SST and PV interneurons. The animals were housed under the standard laboratory lighting condition (light on between 8:00 and 20:00) and temperature (22 ± 1°C) with food and water *ad libitum*. All experiments were conducted following the guidelines for the care and use of animals provided by the National Institutes of Health. All protocols were approved by the Experimental Animal Ethics Review Committee of Southwest University in Chongqing, China.

### 2.2. Forced swim test

In this study, we used both male and female C57BL/6J mice for behavioral testing, ranging in age from 40 to 60 days (equivalent to juvenile to young adulthood). Scopolamine (3 mg/kg) was administered intraperitoneally to the mice 60 min prior to the forced swim test. The test was conducted in a cylindrical glass tank (30 cm high, 20 cm in diameter) filled with 12.5 cm of water at a temperature of 24 ± 1°C. Immobility was defined as the animal floating motionlessly or making only the movements necessary to keep its head above water. Each test lasted 6 min and was recorded using the EthoVision XT video tracking system (Noldus Information Technologies, Leesburg, VA, USA). Immobility time was counted between 2 and 6 min.

### 2.3. Immunohistochemistry and image

Immunohistochemistry was done in 3 SST-Cre/Ai9 and 3 PV-Cre/Ai9 mice of either sex aged P45 to P60. They were deeply anesthetized with isoflurane inhalation and perfused transcardially with the normal saline solution (0.9% NaCl) followed by 4% paraformaldehyde in 0.02 M PBS. Following perfusion, the mice were decapitated, and the brains were removed. After the brains were left overnight in the 4% paraformaldehyde in 0.02 M PBS at 4°C, they were cryoprotected in 30% sucrose in PBS, then rapidly frozen by immersion in 2-methylbutane chilled on dry ice, and the cryostat coronal sections containing the ACC (25 μm) were collected (Leica CM1950, Leica Biosystems Inc., IL).

Sections were incubated in PBS containing 10% methanol and 3% H_2_O_2_ for 1 h to remove endogenous peroxidase activity. After washing in PBS with 0.3% Triton X-100, the sections were incubated in PBS containing 0.3% Triton X-100 and 10% bovine serum albumin (BSA) for 2 h to block non-specific binding and give cell membranes more permeability. The sections were then incubated with primary antibodies in PBS with 0.3% Triton X-100 and 10% BSA. The primary antibodies used in this study were: rat polyclonal anti-somatostatin antibody (1:50, ab30788, Abcam), and mouse monoclonal anti-parvalbumin antibody (1:500, mab354, Millipore). After overnight incubation at 4°C, the sections were washed using PBS with 0.3% Triton X-100 and incubated with second antibodies for 2 h at room temperature. The second antibodies were: donkey anti-rat (1:500, Yeasen, 34412ES60), and goat anti-mouse (1:500, Yeasen, 33212ES60) IgG (H + L), all conjugated to Alexa Fluor 488 (green, Sigma) at a dilution of 1:500 in PBS containing 0.3% Triton X-100 and 10% BSA. The sections were washed in PBS with 0.3% Triton X-100 and mounted on slides, cover-slipped with a mounting medium (Solarbio, S2110, China).

Brain sections of two transgenic mice were observed using a fluorescence microscope (OLYMPUS, BX51) with 4 × (Plan N, N/A 0.10) and 20 × (Plan N, N/A 0.4) lenses. The filter parameters used were BP460–495 nm, DM505 nm, BA510–550 nm for Alexa Fluor, and BP510–550 nm, DM570 nm, BA590 nm for Ai9. The images were taken and analyzed using CellSens Standard image analysis software (Olympus Corporation, Japan). Further imaging of immunostaining was performed using a laser confocal microscope (Nikon A1R-Si), with slices scanned at a *Z*-axis thickness of 1 μm and excited by lasers with wavelengths of 488 and 550 nm for green and red fluorescence, respectively. The images were processed using NIS-Elements C image analysis software.

### 2.4. Electrophysiology

#### 2.4.1. Slice preparation

SST-cre/Ai9 and PV-cre/Ai9 mice of both male and female aged P30 to P50 were anesthetized by isoflurane inhalation, decapitated, and the brains were rapidly removed. 350 μm thick coronal slices containing the ACC (+ 1.42 to + 0.38 mm to the bregma (32) were cut using a vibrating microtome (VT1000s, Leica Microsystems, Deerfield, IL, USA) in an ice-cold sucrose-based cutting solution containing (in mM): 210 Sucrose, 26 NaHCO_3_, 1.25 NaH_2_PO_4_, 2.5 KCl, 1 CaCl_2_, 6 MgCl_2_, 20 D-glucose, gassed with 95% O_2_–5% CO_2_ giving a pH of 7.4. The slices were immediately moved to an incubator with artificial cerebrospinal fluid (ACSF) containing (in mM) 124 NaCl, 26 NaHCO_3_, 1.25 NaH_2_PO_4_, 2.5 KCl, 2 CaCl_2_, 2 MgCl_2_, and 10 D-glucose gassed with 95% O_2_ to 5% CO_2_ at 36°C and incubated for 30 to 45 min. Then the slices were maintained at room temperature (22°C ± 0.5). For recording, a single slice was transferred to a submerged recording chamber after at least 1 h of incubation at room temperature and perfused with a continuous flow (1.5 to 2 mL/min) of the ACSF with 2 mM CaCl_2_ and 2 mM MgCl_2_ at the temperature of 31 to 32°C (TC-344C, Warner instruments, Hamden, CT, USA).

#### 2.4.2. Whole-cell recordings

Whole-cell recordings were made from RFP neurons in layers II-III of the ACC using a Heka EPC10 amplifier (HEKA Instruments, Germany). RFP neurons were first identified under fluorescence and then recorded under infrared differential interference contrast (IR-DIC) video microscopy with a fixed stage microscope (Axioskop-FS; Olympus BX51WI, Japan) equipped with a 40 X, 0.8 W water-immersion objective and Orca-spark Digital CMOS camera (C11220-36U, Hamamatsu, Japan). Patch pipettes were pulled with a puller (P-97, Sutter Instruments, Novato, CA, USA) from Wiretrol II capillary glass (Drummond Scientific, Broomall, PA, USA) and had a resistance of 3-5 MΩ after being filled with internal solutions. The internal solution for recording excitatory synaptic responses contained (in mM): 125 K-gluconate, 8 NaCl, 10 HEPES, 2 MgATP, 0.3 Na_3_GTP, 0.2 EGTA, 0.1% biocytin (pH = 7.2 with KOH, osmolarity = 290–300 mOsM). The 125 mM KCl-based internal solution was used to record inhibitory synaptic responses (in mM): 125 KCl, 8 NaCl, 10 HEPES, 2 MgATP, 0.3 Na_3_GTP, 0.2 EGTA, 0.1% biocytin (pH = 7.2 with KOH, osmolarity = 290–300 mOsM). Spontaneous excitatory postsynaptic currents (sEPSCs) were recorded at a holding potential of −65 mV at a voltage-clamp mode. To block GABAergic synaptic activity, picrotoxin (50 μM) was added to the bath solution. Spontaneous inhibitory postsynaptic currents (sIPSCs) were recorded with the pipettes filled with KCl-based internal solution at −65 mV. 2-amino-5-phosphonopentanoic acid (APV, 50 μM, Tocris) and 2, 3-dihydroxy-6-nitro-7-sulfamoyl-benzo (F) quinoxaline (NBQX, 10 μM, Sigma) were added in the bath solution to block excitatory synaptic activities. Tetrodotoxin (TTX, 1 μM, Tocris) was added in addition for recording miniature EPSCs (mEPSCs) and miniature IPSCs (mIPSCs). Evoked EPSCs and IPSCs were induced with paired-pulse (20 Hz) stimulations by patch pipettes filled with ACSF (3–6 MΩ) placed about 50 μm away from the thick shafts of the dendrites of RFP neurons. The place of stimulating pipettes was carefully adjusted not to evoke polysynaptic responses. The series resistance was 14–20 MΩ, and the recordings were discarded if a change of series resistance above 20% occurred. All experiments were carried out at 31 to 32°C (TC-344C, Warner instruments).

#### 2.4.3. Data acquisition

Electrophysiological data were acquired using PatchMaster V2 × 80 (HEKA Electronik, Germany). The signals were digitized at 10 kHz and filtered at 2 kHz. Offline analysis was performed with Clampfit 10 (Molecular Devices, San Jose, CA, USA), MiniAnalysis software (Synaptosoft Inc., NJ, USA), and Origin 8 (OriginLab, Northampton, MA, USA). The frequency and amplitude of sEPSCs, mEPSCs, sIPSCs, and mIPSCs were analyzed with MiniAnalysis software. Synaptic events were first detected with parameters optimized for each cell and then visually confirmed before analysis. The amplitude of evoked EPSCs and IPSCs was measured with Clampfit 10, and paired-pulse plasticity was valued as the ratio of the amplitude of the second response to the first response.

### 2.5. Statistical analysis

Data were analyzed using GraphPad Prism 8.0 software (GraphPad Software, La Jolla, CA, USA) and expressed as mean ± standard error (mean ± SEM). The Kolmogorov-Smirnov (K-S) test was used to test the normality of data, and the F test for the homogeneity of the variance. Paired t-test or repeated one-way ANOVA test (RM one-way ANOVA) with Tukey’s *post hoc* multiple comparisons test was used to compare data with a normal distribution. Wilcoxon matched-pairs signed-rank test (Wilcoxon test) or Friedman test with Dunn’s *post hoc* multiple comparisons test was used to compare data that are not in the normal distribution. The statistical significance is defined as *p* < 0.05. Detailed statistics are reported in Supplementary Table 1.

### 2.6. Chemicals

All drugs were first dissolved in distilled water as stock solutions and diluted to desired concentrations before use. They included muscarine 10 mM (Tocris Cookson Ltd., Bristol, UK), scopolamine 1 mM (Sigma, St. Louis, MO, USA), picrotoxin 5 mM (Tokyo chemical industry Co., Ltd., Tokyo, Japan), DL-2-amino-5-phosphonopentanoic acid 50 mM (Tocris Cookson Ltd., Bristol, UK), NBQX disodium salt hydrate 10 mM (Sigma, St. Louis, MO, USA), and tetrodotoxin 1 mM (TTX, Tocris Cookson, Bristol, UK).

## 3. Results

**FIGURE 1:**
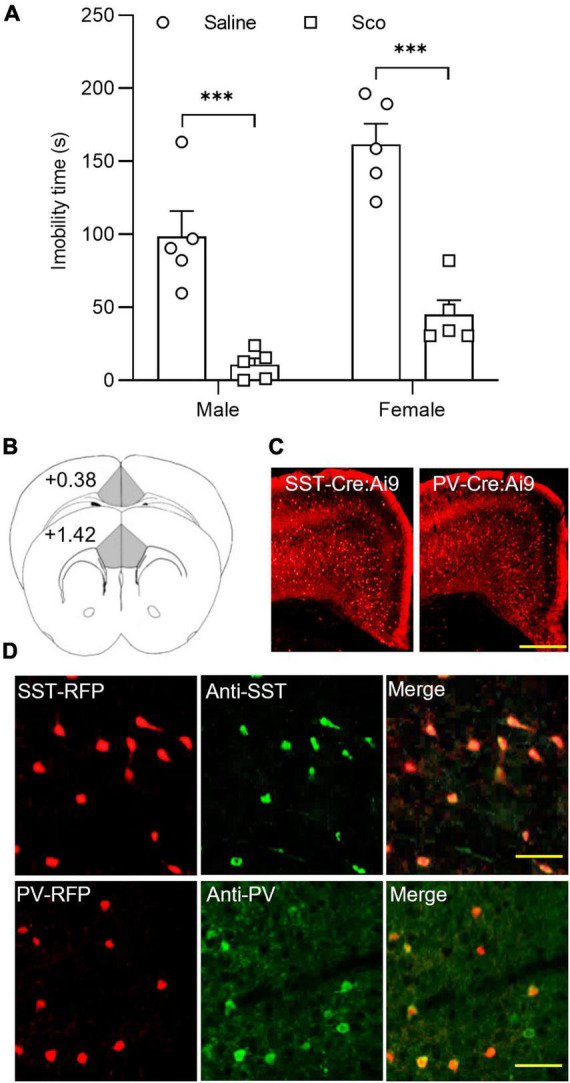
The effects of scopolamine on the forced swim test and RFP-expressing neurons in the ACC of SST-cre/Ai9 and PV-cre/Ai9 mice. **(A)** Plots show the effect of scopolamine injection (3 mg/Kg, i.p.) on the immobility time of the forced swim test (FST) in male (P40-60, *n* = 5 for each group, left panel) and female C57BL/6J mice (P40-60, *n* = 5 for each group, right panel). Control mice were injected the same volume of saline. The FST was performed 60 min after injection. **(B)** A diagram illustrates the location of the ACC in coronal sections of the mouse brain. **(C)** Fluorescent images show the distribution of red fluorescent protein (RFP) positive neurons in the anterior cingulate cortex (ACC) from an SST-cre/Ai9 mouse (left) and a PV-cre/Ai9 mouse (right). Scale bar: 100 μm. **(D)** Immunohistochemistry shows the staining of RFP neurons to SST antibody (green) in an SST-cre/Ai9 mouse brain section (upper panel) and PV antibody (green) in a PV-cre/Ai9 mouse brain section. Scale bar: 50 μm. Scop, scopolamine; ACC, anterior cingulate cortex; SST, somatostatin; PV, parvalbumin; RFP, red fluorescent protein. ***p < 0.001.

First, we tested the effects of the non-selective mAChR antagonist, scopolamine, on the forced swim test (FST) in C57BL/6J male and female mice. As illustrated in Figure 1A, compared to control mice treated with saline, scopolamine (3 mg/Kg, i.p.) significantly reduced immobility time in both male and female mice [*F*(1,16) = 68.04, *p* < 0.0001, *n* = 5 for each group, Two-way ANOVA; *post-hoc* test: Male Saline/Sco, *p* = 0.0003; Female Saline/Sco, *p* < 0.0001]. These data indicate that scopolamine reduces immobility time in both male and female mice.

We then employed SST-Cre/Ai9-RFP and PV-Cre/Ai9-RFP mice for examining mAChR modulation of synaptic activity in SST and PV interneurons within the ACC. These two transgenic mice allowed us to easily identify SST and PV interneurons (Figures 1B–D). Our previous work has characterized the electrophysiological properties of RFP-positive neurons in the ACC using the whole-cell recording method in both types of mice (33). The properties of RFP neurons in SST- and PV-Cre/Ai9-RFP mice are consistent with that of SST and PV interneurons reported in other studies, respectively (34, 35).

### 3.1. Activation of mAChRs enhances spontaneous excitatory synaptic activities in both SST and PV interneurons in the ACC

We first examined the effect of mAChR activity on the excitatory synapses of SST and PV interneurons in the ACC. Whole-cell recordings were performed to record excitatory synaptic activities, including sEPSCs, mEPSCs, and evoked EPSCs. Muscarine (10 μM), a non-selective mAChR agonist, and scopolamine (100 nM), a non-selective mAChR antagonist, were used to activate and block mAChRs, respectively. The drugs were applied in the bath solution after a baseline recording of 5 to 10 min. The same strategy was used in the experiments for the inhibitory synaptic activity.

**FIGURE 2:**
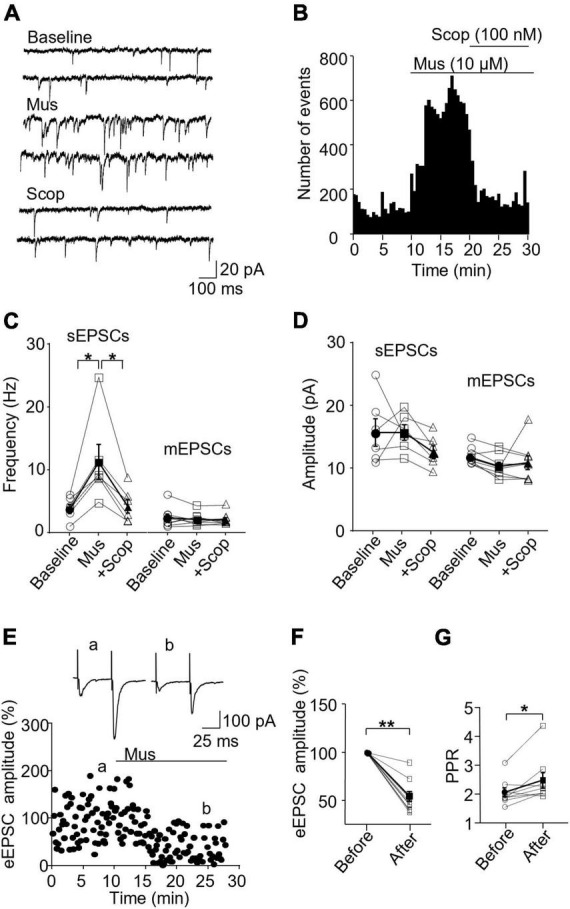
Activation of mAChRs enhances spontaneous excitatory synaptic activity in SST interneurons in the ACC. **(A)** Representative traces of sEPSCs from an SST interneuron in the ACC before and after application of muscarine (Mus, 10 μM) and subsequent addition of scopolamine (Scop, 100 nM). **(B)** A histogram from an SST interneuron shows the effect of bath application of muscarine and subsequent addition of scopolamine on sEPSC frequency. **(C,D)** Summarized data show the effect of muscarine on sEPSC (*n* = 6 cells/6 mice) and mEPSC (*n* = 6 cells/3 mice) frequency and amplitude, respectively. **(E)** A representative experiment shows the effect of muscarine on evoked EPSCs (*n* = 9 cells/6 mice). Insets: evoked EPSCs traces taken at the times marked by a and b in the graph. **(F,G)** Summarized data show the effect of muscarine on evoked EPSC amplitude and the paired-pulse ratio, respectively (PPR, the 2nd EPSC/the 1st EPSC in amplitude). *p < 0.05, **p < 0.01. Mus, muscarine; Scop, scopolamine.

In SST interneurons, we observed that muscarine significantly increased sEPSC frequency, which was reversed by scopolamine [*F*(1.072,5.360) = 10.96, *p* = 0.018, *n* = 6 cells/6 mice, RM one-way ANOVA; *post-hoc* test: baseline/muscarine, *p* = 0.028; baseline/scopolamine, *p* > 0.9999, Figures 2A–C and Supplementary Figure 3A]. Averaged sEPSC frequencies before and 10 min after muscarine were 3.97 ± 0.73 Hz and 11.41 ± 2.82 Hz, respectively. The amplitude, however, remained unchanged [*F*(1.205,6.027) = 1.747, *p* = 0.241, RM one-way ANOVA, *n* = 6 cells/6 mice, Figure 2D and Supplementary Figure 3A], 15.74 ± 2.16 pA before and 15.75 ± 1.22 pA 10 min after muscarine. We noticed that muscarine induced an inward current of 21.56 ± 6.42 pA during the sEPSC recording. We next examined muscarine’s effect on mEPSCs induced by spontaneous transmitter release from presynaptic terminals independent of action potentials. We observed that muscarine had no effect on mEPSC frequency [*F*(1.456,10.19) = 0.7260, *p* = 0.465, *n* = 6 cells/3 mice, RM one-way ANOVA, Figure 2C] and amplitude [*F*(1.193,8.349) = 1.405, *p* = 0.28, *n* = 6 cells/3 mice, RM one-way ANOVA, Figure 2D]. mEPSC frequencies before and after muscarine were 2.41 ± 0.57 and 2.13 ± 0.35 Hz, respectively. And mEPSC amplitudes before and after muscarine were 11.59 ± 0.49 and 10.11 ± 0.66 pA, respectively. Muscarine, however, significantly reduced the amplitude of the evoked EPSCs (*p* = 0.004, Wilcoxon test, *W* = −45, *n* = 9 cells/6 mice Figures 2E, F), which was accompanied by a significant increase in paired-pulse ratio (*p* = 0.019, Wilcoxon test, *W* = 39, Figure 2G).

**FIGURE 3:**
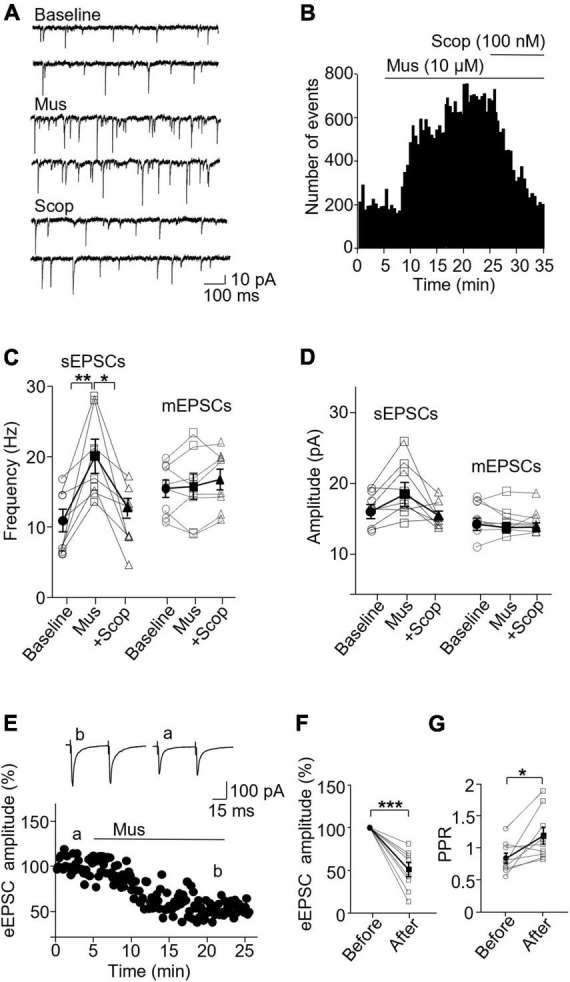
Activation of mAChRs enhances spontaneous excitatory synaptic activity in PV interneurons in the ACC. **(A)** Representative traces of sEPSCs from a PV interneuron in the ACC before and after application of muscarine and scopolamine. **(B)** A histogram shows the effect of muscarine and scopolamine on sEPSC frequency in the PV interneuron. **(C,D)** Summarized data show the effect of muscarine on sEPSC (*n* = 8 cells/6 mice) and mEPSC (*n* = 8 cells/5 mice) frequency and the amplitude. **(E)** A representative experiment shows the effect of muscarine on evoked EPSCs. Insets: evoked EPSCs traces taken at the times marked by a and b in the graph. **(F,G)** Summarized data show the effect of muscarine on evoked EPSC (*n* = 8 cells/5 mice) amplitude and paired-pulse ratio, respectively. *p < 0.05, **p < 0.01, and ***p < 0.001.

In PV interneurons, muscarine also significantly enhanced sEPSC frequency, from 10.65 ± 1.57 Hz in the baseline to 19.89 ± 1.86 Hz 10 min after muscarine, which was reversed by scopolamine (baseline/muscarine, *p* = 0.004; baseline/scopolamine, *p* = 0.952; Friedman test, *F* = 13, *n* = 8 cells/6 mice, Figures 3A–C and Supplementary Figure 3B). The amplitude was not altered [*F*(1.122,7.851) = 15.65, *p* = 0.355, *n* = 8 cells/6 mice, RM one-way ANOVA, Figure 3D and Supplementary Figure 3B], 16.10 ± 0.87 and 18.41 ± 1.49 pA before and after muscarine, respectively. Muscarine did not alter mEPSCs frequency [*F*(1.722,12.06) = 1.238, *p* = 0.317, *n* = 8 cells/5 mice, RM one-way ANOVA, Figure 3C] and amplitude [*F*(1.169,8.182) = 0.363, *p* = 0.596, *n* = 8 cells/5 mice, RM one-way ANOVA, Figure 3D]. mEPSC frequencies before and after muscarine were 15.51 ± 1.26 and 15.76 ± 1.82 Hz, respectively. mEPSC amplitudes before and after muscarine were 15.26 ± 0.85 and 14.81 ± 0.69 pA. Muscarine significantly reduced the amplitude of the evoked EPSCs [*t*(7) = 5.894, *p* = 0.001, paired t-test, *n* = 8 cells/5 mice, Figures 3E, F], which was accompanied by a significant increase in the paired-pulse ratio [*t*(7) = 2.938, *p* = 0.022, paired t-test, Figure 3G]. We also observed that muscarine elicited inward currents (19.64 ± 2.67 pA in sEPSC recordings, and 24.69 ± 3.47 pA in mEPSC recordings).

These data indicate activation of mAChRs by muscarine non-specifically increases spontaneous excitatory transmission in both SST and PV interneurons, mainly in a pre-synaptic manner. Moreover, scopolamine can completely block muscarine’s potentiation effects.

### 3.2. Blocking mAChRs does not affect excitatory synaptic activity to SST and PV interneurons

To test whether mAChRs are tonically active and modulate the excitatory synaptic activity in SST and PV interneurons, we examined the effect of scopolamine on the excitatory synaptic activity in two interneuron subtypes. In SST interneurons, the application of scopolamine (100 nM) had no significant effect on sEPSC frequency and amplitude (Supplementary Figures 1A–D). The frequencies before and after scopolamine were 8.09 ± 2.20 and 7.69 ± 2.55 Hz, respectively (*p* = 0.688, *n* = 7 cells/7 mice, Wilcoxon test, *W* = −6), and the amplitudes were 17.12 ± 2.04 pA and 15.14 ± 1.95 pA, respectively (*p* = 0.078, Wilcoxon test, *W* = −22). Scopolamine also did not affect mEPSC frequency and amplitude (Supplementary Figures 1C, D). mEPSC frequencies before and after perfusion were 6.00 ± 2.35 and 6.19 ± 2.43 Hz, respectively (*p* = 0.688, *n* = 7 cells/4 mice, Wilcoxon test, *W* = 6), and the amplitudes were 10.29 ± 0.55 and 10.19 ± 0.59 pA, respectively [*t*(6) = 0.305, *p* = 0.771, paired *t*-test]. Likewise, scopolamine did not affect the evoked EPSCs [*t*(10) = 1.664, *p* = 0.127, *n* = 11 cells/6 mice, paired *t*-test, Supplementary Figures 1E, F] and paired-pulse ratio [*t*(10) = 1.434, *p* = 0.182, paired t-test, Supplementary Figure 1G].

In PV interneurons, scopolamine also did not affect the frequency and amplitude of sEPSCs [frequency: *t*(6) = 1.503, *p* = 0.184; amplitude: *t*(6) = 0.237, *p* = 0.821, *n* = 7 cells/6 mice, paired t-test, Supplementary Figures 2A–D] and mEPSCs [frequency: *t*(5) = 0.725, *p* = 0.501, paired t-test; amplitude: *p* = 0.58, *n* = 7 cells/5 mice, Wilcoxon test, *W* = 8, Supplementary Figures 2C, D]. sEPSC frequency was 15.80 ± 1.34 Hz before and 16.81 ± 1.64 Hz after scopolamine, and the amplitudes were 13.15 ± 0.62 and 13.07 ± 0.63 pA. mEPSC frequency was 11.10 ± 0.69 Hz before and 10.82 ± 0.72 Hz after scopolamine, and the amplitudes were 13.15 ± 0.62 and 13.07 ± 0.63 pA. Scopolamine did not affect the evoked EPSCs [*t*(7) = 1.548, *p* = 0.166, *n* = 8 cells/6 mice, paired t-test, Supplementary Figures 2E, F] and the paired-pulse ratio [*t*(7) = 0.311, *p* = 0.765, paired *t*-test, Supplementary Figure 2G]. This suggests scopolamine inhibition of mAChRs won’t affect the excitatory synaptic transmission to both of these interneurons.

### 3.3. Activation of mAChRs suppresses inhibitory synaptic activity specifically in SST

**FIGURE 4:**
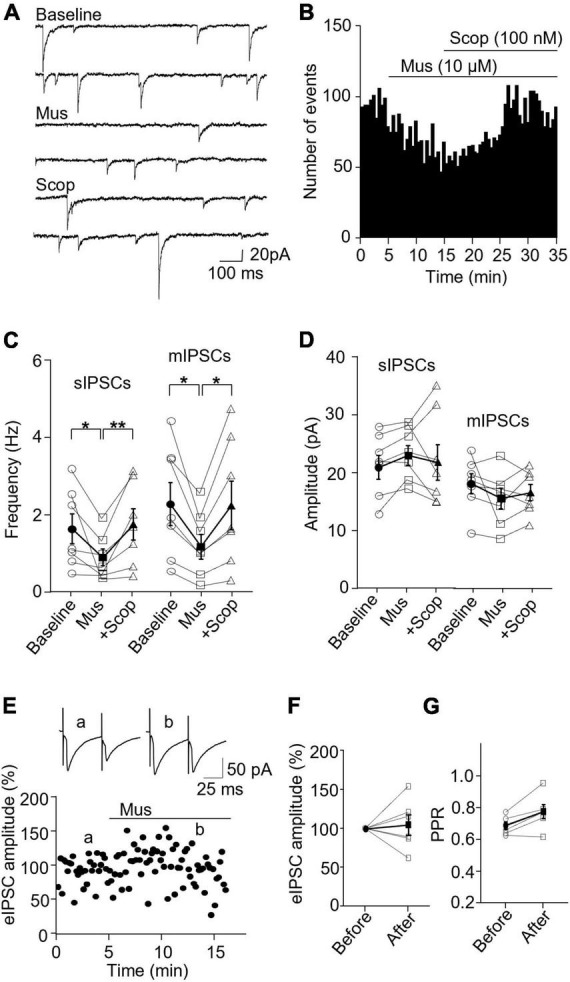
Activation of mAChRs attenuates inhibitory synaptic activity in SST interneurons. **(A)** Representative traces of sIPSCs from an SST interneuron in the ACC before and after bath application of muscarine and scopolamine. **(B)** A histogram shows the effect of muscarine and scopolamine on sIPSC frequency in the SST interneurons. **(C,D)** Summarized data shows the effect of muscarine on sIPSC (*n* = 7 cells/3 mice) and mIPSC (*n* = 7 cells/3 mice) frequency and the amplitude. **(E)** A representative experiment shows the effect of muscarine on evoked IPSCs. Insets: evoked IPSCs traces taken at the times indicated by a and b in the graph. **(F,G)** Summarized data show the effect of muscarine on evoked IPSC amplitude (*n* = 6 cells/5 mice) and the paired-pulse ratio, respectively. *p < 0.05, **p < 0.01.

We then wanted to see how mAChR activity modulates inhibitory synapses in SST and PV interneurons. In SST interneurons, the application of muscarine decreased both sIPSC and mIPSC frequency without any effect on the amplitude (Figures 4A–C). sIPSC frequencies before and after muscarine application were 1.61 ± 0.39 and 0.86 ± 0.22 Hz, respectively, and scopolamine reversed the decrease (*p* = 0.0012, *n* = 7 cells/3 mice, Friedman test; *post hoc*: muscarine vs. baseline: *p* = 0.016, baseline vs. scopolamine: *p* = 0.38). The amplitudes were 21.47 ± 2.04 and 23.51 ± 1.69 pA, respectively [*F*(1.116,6.694) = 0.543, *p* = 0.505, *n* = 7 cells/3 mice, RM one-way ANOVA, Figure 4D]. For mIPSCs, the frequencies before and after muscarine were 2.37 ± 0.56 and 1.26 ± 0.32 Hz, respectively, which was also reversed by scopolamine [*F*(1.738,10.43) = 9.759, *p* = 0.005, *n* = 7 cells/3 mice, RM one-way ANOVA; *post hoc*: muscarine vs. baseline: *p* = 0.025, baseline vs. scopolamine: *p* = 0.984, Figure 4C and Supplementary Figure 3C]. The amplitudes were 18.12 ± 1.73 and 15.57 ± 1.79 pA, respectively [*F*(1.693,10.16) = 1.364, *p* = 0.293, *n* = 7 cells/3 mice, RM one-way ANOVA, Figure 4D and Supplementary Figure 3C]. Muscarine had no significant effect on the evoked IPSCs (*p* = 0.688, *n* = 6 cells/5 mice, Wilcoxon test, *W* = 5, Figures 4E, F) and the paired-pulse ratio (*p* = 0.063, *w* = 19, Wilcoxon test, Figure 4G).

**FIGURE 5:**
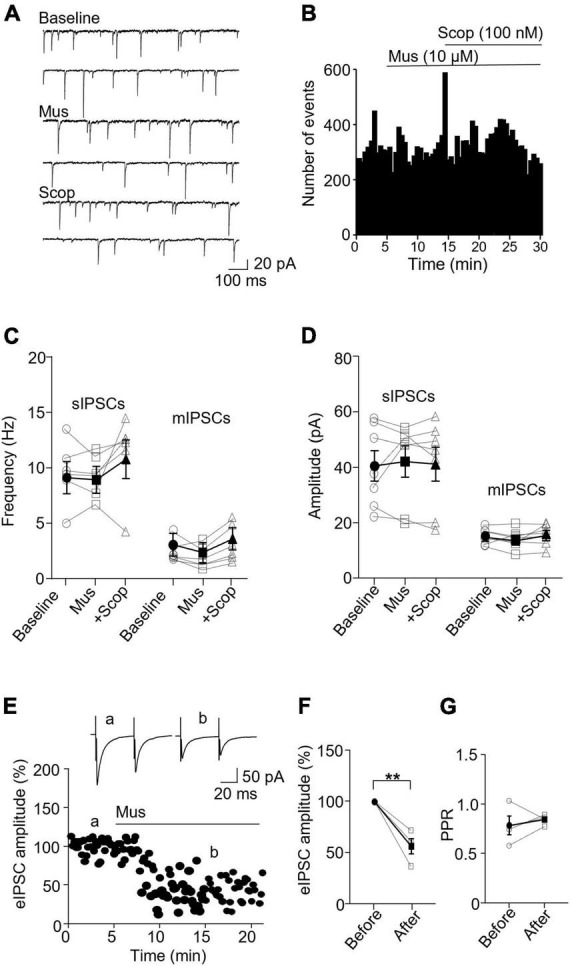
Activation of mAChRs does not affect inhibitory synaptic activity in PV interneurons. **(A)** Representative traces of sIPSCs recorded from a PV interneuron before and after perfusion of muscarine. **(B)** A histogram shows the effect of muscarine and scopolamine on sIPSC frequency in the PV interneuron. **(C,D)** Summarized data show the effect of muscarine on sIPSC (*n* = 6 cells/6 mice) and mIPSC (*n* = 6 cells/5 mice) frequency and amplitude. **(E)** A representative experiment from a PV interneuron shows the effect of muscarine on evoked IPSCs. Insets: evoked IPSCs traces taken at the times marked by a and b in the graph. **(F,G)** Summarized data show the effect of muscarine on evoked IPSC amplitude (*n* = 4 cells/3 mice) and paired-pulse ratio, respectively. **p < 0.01.

In PV interneurons, muscarine had no significant effect on sIPSC and mIPSC frequency and amplitude (Figures 5A–C). sIPSC frequencies before and after muscarine were 9.59 ± 1.13 and 9.33 ± 0.78 Hz, respectively [*F*(1.333,6.664) = 2.467, *p* = 0.162, *n* = 7 cells/6 mice, RM one-way ANOVA], and the amplitudes were 40.52 ± 5.49 pA and 42.12 ± 5.61, respectively (*p* = 0.620, *F* = 1.143, *n* = 7 cells/6 mice, Friedman test, Figure 5D). mIPSC frequencies before and after muscarine were 2.58 ± 0.41 and 2.00 ± 0.40 Hz, respectively [*F*(1.546,7.732) = 6.492, *p* = 0.027, *n* = 7 cells/5 mice, RM one-way ANOVA; *post hoc*: muscarine vs. baseline: *p* = 0.119, baseline vs. scopolamine: *p* = 0.421, muscarine vs. scopolamine: *p* = 0.005, Figure 5C and Supplementary Figure 3D], and the amplitudes were 15.33 ± 1.06 and 14.37 ± 1.29 pA, respectively [*F*(1.930,11.58) = 0.985, *p* = 0.40, *n* = 7 cells/5 mice, RM one-way ANOVA, Figure 5D and Supplementary Figure 3D]. Interestingly, muscarine significantly reduced the amplitude of evoked IPSCs in PV interneurons [*t*(3) = 5.948, *p* = 0.010, paired *t*-test, *n* = 4 cells/3 mice, Figures 5E, F] with no changes in the paired-pulse ratio [*t*(3) = 0.711, *p* = 0.528, paired *t*-test, Figure 5G]. The above data demonstrate muscarine activation of mAChRs selectively reduces inhibitory synaptic transmission, both sIPSC and mIPSC frequency in SST interneurons, mainly in a pre-synaptic manner.

### 3.4. Scopolamine enhances inhibitory synaptic transmission specifically in SST interneurons

**FIGURE 6:**
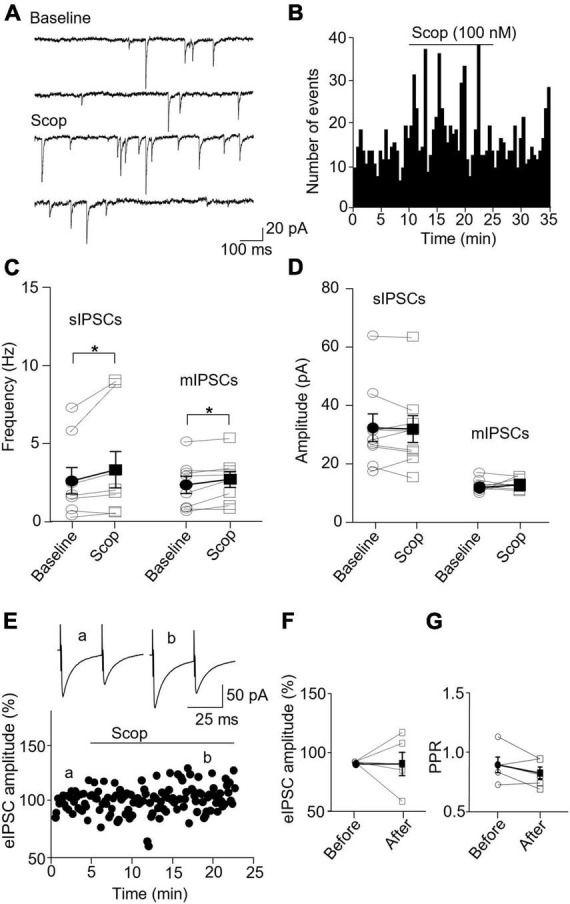
Blocking mAChRs enhances inhibitory synaptic transmission in the SST interneuron. **(A)** Representative traces of sIPSCs from an SST interneuron before and after application of scopolamine. **(B)** A histogram shows the effect of scopolamine on sIPSC frequency in the SST interneuron. **(C,D)** Summarized data show the effect of scopolamine on sIPSC (*n* = 7 cells/5 mice) and mIPSC (*n* = 8 cells/5 mice) frequency and amplitude, respectively. **(E)** A representative experiment from an SST interneuron shows the effect of scopolamine on the amplitude of evoked IPSCs. Inserts: the traces of evoked IPSCs taken at the times indicated by a and b in the graph. **(F,G)** Summarized data showing the effect of scopolamine on evoked IPSC amplitude (*n* = 5 cells/4 mice) and paired-pulse ratio, respectively. *p < 0.05.

In SST interneurons, bath application of scopolamine significantly increased sIPSC and mIPSC frequency without altering the amplitude (Figures 6A–D). sIPSC frequencies before and after scopolamine were 2.89 ± 1.00 Hz and 3.80 ± 1.39, respectively (*p* = 0.031, *n* = 7 cells/5 mice, Wilcoxon test, *W* = 26), and the amplitudes were 32.29 ± 4.74 and 31.91 ± 4.61 pA, respectively (*p* = 0.73, Wilcoxon test, *W* = −7). For mIPSCs, the frequencies before and after scopolamine were 2.37 ± 0.55 and 2.72 ± 0.52 Hz, respectively [*t*(7) = 2.593, *p* = 0.036, *n* = 8 cells/5 mice, paired *t*-test, Figure 6C], and the amplitudes were 12.65 ± 0.79 and 13.34 ± 0.69 pA, respectively [*t*(7) = 0.716, *p* = 0.497, *n* = 8 cells/5 mice, paired t-test, Figure 6D]. Scopolamine did not change the amplitude of evoked IPSCs [*t*(4) = 0.0199, *p* = 0.99, paired *t*-test, Figures 6E, F] and paired-pulse ratio [*t*(4) = 1.486, *p* = 0.212, *n* = 5 cells/4 mice, paired *t*-test, Figure 6G].

**FIGURE 7:**
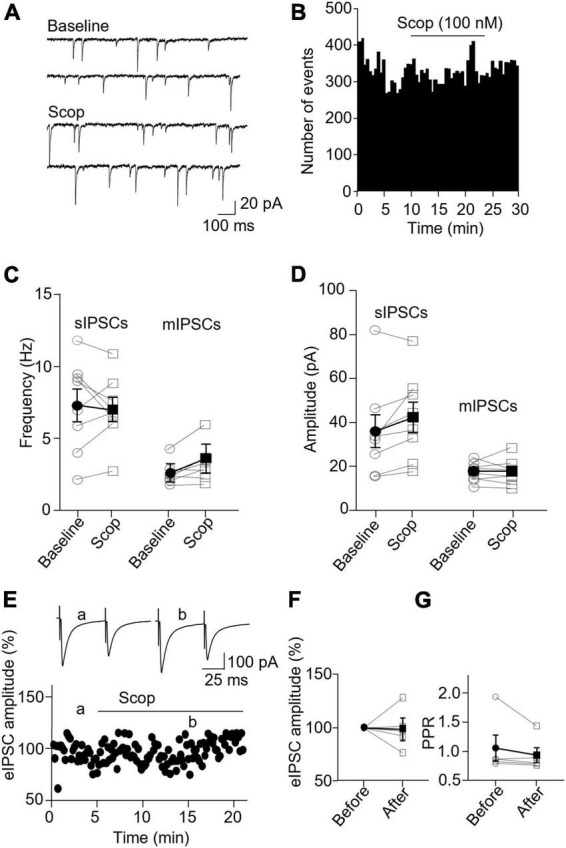
Blocking mAChRs does not affect inhibitory synaptic transmission in PV interneurons. **(A)** Representative traces of sIPSCs from a PV interneuron before and after application of scopolamine. **(B)** A histogram shows the effect of scopolamine on sIPSC frequency in the PV interneuron. **(C,D)** Summarized data show the effect of scopolamine on sIPSC (*n* = 8 cells/6 mice) and mIPSC (*n* = 8 cells/6 mice) frequency and amplitude, respectively. **(E)** A representative experiment from a PV interneuron shows the effect of scopolamine on the amplitude of evoked IPSCs. Inserts: the traces of evoked IPSCs taken at the time indicated by a and b in the graph. **(F,G)** Summarized data show the effect of scopolamine on evoked IPSCs amplitude (*n* = 5 cells/4 mice) and paired-pulse ratio, respectively.

In PV interneurons, scopolamine had no significant effect on sIPSC and mIPSC frequency and amplitude (Figures 7A–D). sIPSC frequencies before and after scopolamine were 7.31 ± 1.13 and 7.05 ± 0.83 Hz, respectively [*t*(7) = 0.396, *p* = 0.704, *n* = 8 cells/6 mice, paired *t*-test], and the amplitudes were 36.02 ± 7.55 and 42.49 ± 6.90 pA, respectively [*t*(7) = 2.274, *p* = 0.057, *n* = 8 cells/6 mice, paired *t*-test]. For mIPSCs, the frequencies before and after scopolamine were 2.62 ± 0.31 and 3.29 ± 0.50 Hz, respectively [*t*(7) = 2.274, *p* = 0.051, *n* = 8 cells/5 mice, paired *t*-test, Figure 7C], and the amplitudes were 17.49 ± 1.57 and 17.45 ± 2.03 pA, respectively [*t*(7) = 0.033, *p* = 0.970, paired *t*-test, Figure 7D]. Scopolamine did not change the amplitude of evoked IPSCs and paired-pulse ratio [evoked IPSCs: *t*(4) = 0.116, *p* = 0.914, *n* = 5 cells/4 mice, paired t-test; paired-pulse ratio: *t*(4) = 1.306, *p* = 0.262, *n* = 5 cells/4 mice, paired *t*-test, Figures 7E–G]. This reveals scopolamine inactivation of mAChRs selectively enhances the presynaptic GABA release to SST interneurons.

## 4. Discussion

In this study, we have characterized mAChR modulation of excitatory and inhibitory synapses in SST and PV interneurons in the ACC. We observed that mAChR activation by muscarine remarkably enhanced sEPSC frequency in both SST and PV interneurons. The enhancement was likely due to the increased firing of presynaptic pyramidal neurons since sEPSC amplitude and mEPSC frequency and amplitude remained unchanged. Scopolamine, which blocks mAChR activity, completely reversed the effect of muscarine but did not affect sEPSCs and mEPSCs in both interneuron subtypes when applied alone. We further observed that sIPSC and mIPSC frequency were significantly reduced by muscarine and enhanced by scopolamine in SST, but not PV, interneurons. Collectively, our results revealed that mAChR activation modulates synaptic activity in neuron and synapse type-specific manners. The enhancement of inhibitory synaptic activity in the SST interneuron by scopolamine may further disinhibit the pyramidal neurons and, thus, may contribute to the initial mechanisms of scopolamine’s rapid antidepressant action.

Activation of mAChRs results in diverse effects on neuronal properties dependent on neuron types and brain regions (36–41). mAChRs also heterogeneously modulate synaptic activities (42–44). These various effects of mAChRs are thought to be due to differential expressions of mAChR subtypes. mAChRs comprise five subtypes (M1 to M5) that are heterogeneously expressed and distributed in the excitatory and GABAergic inhibitory neurons in the CNS, and each subtype may have unique functions (21, 45–47). mAChR modulation of synaptic activities has been studied extensively in mPFC and the hippocampus. However, much less has been done in the ACC, a region of the PFC that intensively and reciprocally connects with other brain regions and has been indicated to play an essential role in depression and antidepressant action (48–50). We found that mAChR activation enhanced sEPSC frequency in both SST and PV interneurons but left sEPSC amplitude and mEPSC frequency and amplitude unaffected. Our result is thus consistent with the previous finding that mAChR activation depolarizes and enhances the firing of the excitatory neurons (36, 51). In line with other studies, our study also showed that activation of mAChRs remarkably depressed the excitatory synaptic responses evoked by stimulating the presynaptic fibers (38). A presynaptic mechanism likely mediates the reduction because the reduction was accompanied by enhanced paired-pulse facilitation, a presynaptic event (52, 53). The reduction of evoked synaptic transmission by mAChR activation has been widely reported in pyramidal neurons in mPFC, hippocampus, and other regions (43, 44, 54).

The direct effect of antagonizing mAChRs on the synaptic activity in pyramidal and interneurons has not been extensively studied. Since the discovery of the rapid antidepressant action of scopolamine, mAChR modulation on the function of interneurons has been targeted for understanding the underlying mechanisms. We found that scopolamine alone did not affect the spontaneous and evoked EPSCs but reversed muscarine’s effect. The result implies that the tonic mAChR activity might be low due to a low acetylcholine concentration or a low expression of mAChRs at the excitatory synapses. It is also possible that the slice preparation dilutes and hence reduces the local acetylcholine concentration. *In vivo* study may overcome the drawbacks of slice preparation and reveal possible tonic mAChR activity and its modulation of neuronal activities.

Our results revealed that mAChR activation differently modulated inhibitory synapses between SST and PV interneurons. We found that mAChR activation decreased both sIPSC and mIPSC frequency in SST interneurons but not in PV interneurons. Our finding is in contrast to other studies showing that mAChR activation enhanced sIPSC frequency in pyramidal neurons (55–57) and interneurons (38) in different brain regions (58, 59). We do not have immediate explanations for the discrepancy. It may indicate that mAChR modulation of inhibitory synapses is specific to targeted neuron types and brain regions. In support of this notion, activation of mAChRs was reported to suppress mIPSCs in glutamatergic neurons in the superior colliculus and dopamine neurons in the midbrain (60, 61). We noticed that mAChR activation-induced inward currents in both SST and PV interneurons, which could lead to an increase in excitability and firing in both interneuron subtypes. We expected mAChR activation could increase sIPSC frequency, but instead, we observed a decrease in sIPSC frequency in SST interneurons and no apparent change in PV interneurons. It is noteworthy to clarify the underlying mechanism in a future study. We also observed that mAChR activation affected evoked ISPCs differently in SST and PV interneurons. Activation of mAChRs remarkably reduced the evoked IPSCs in PV interneurons without significant effect in SST interneurons. More experiments are required to confirm the finding and reveal the mechanisms. The different responses to mAChRs in between SST and PV interneurons are also supported by other studies. SST interneurons were found to express more mAChRs than PV interneurons (21) and yield stronger response to mAChR activation (56, 62–65). Together, these results indicate that SST and PV interneurons are differently modulated by mAChRs, which may mediate different physiological functions and pathological processes.

Scopolamine significantly enhanced both sIPSCs and mIPSCs frequency in SST interneurons, indicating that inhibitory synaptic activity in SST interneurons is modulated by a tonic activity of mAChRs. Such modulation was not found in the inhibitory synapse in PV interneurons and excitatory synapses in both interneuron subtypes. Enhanced inhibitory synaptic activity in SST interneurons may increase the excitability of targeted pyramidal neurons. Thus, our results support the disinhibition hypothesis that has been proposed to underlie the initial phase of scopolamine’s rapid antidepressant action (21, 66, 67). However, the interpretations of our results should be taken with caution. Given the nature of the brain slice preparation, the neuronal circuitries are only partially preserved and the environment around neurons and synapses is altered in the brain slices, which may affect the endogenous activity of receptors and synapses. Thus, these results may not entirely apply to the intact brain. A future *in vivo* study is, therefore, needed to further unravel the effect of mAChR activity on different subtypes of interneurons. Furthermore, future studies should aim to identify the specific synaptic targets of scopolamine on SST and PV interneurons. Additionally, non-pharmacological methods such as optogenetics or chemogenetics could be used to modulate these targets, providing insight into the mechanisms underlying scopolamine’s antidepressant effects.

One limitation of this study is the lack of consideration for sex differences. It is known that sex-specific neurobiological processes and neuronal circuits may contribute to the observed differences in depression between males and females (68). Furthermore, previous research has shown that scopolamine has a larger antidepressant effect in women than in men (8). While our results did not show any significant differences in the effects of scopolamine on FST between male and female mice, it is still possible that sex plays a role in the mechanisms underlying its antidepressant action. Another limitation of this study is that it did not examine the effect of scopolamine on animal models of depression. It is unclear whether the inhibitory synaptic properties of SST interneurons are altered in depression and whether scopolamine can reverse these changes. Further research is necessary to address these questions.

In summary, the present study investigated mAChR modulation of synaptic activities in SST and PV interneurons in the ACC. We show that mAChR activation enhanced spontaneous excitatory synaptic activities in both SST and PV interneurons but selectively attenuated spontaneous inhibitory synaptic activity in SST interneurons. Scopolamine also selectively enhanced inhibitory synaptic activity in SST interneurons, likely leading to the disinhibition of pyramidal neurons. Our results suggest that scopolamine’s rapid antidepressant action may be related to its impact on synaptic mechanisms.

## Data availability statement

The original contributions presented in this study are included in this article/Supplementary material, further inquiries can be directed to the corresponding authors.

## Ethics statement

The animal study was reviewed and approved by Experimental Animal Ethics Review Committee of Southwest University.

## Author contributions

TH, ML, CW, WW, and BY performed the electrophysiological experiments, analyzed the data, and wrote the manuscript. CG, JH, LC, and PG performed the histology and analyzed the data. YY, HC, and TT the designed the experiments, supervised the project, and revised the manuscript. All authors contributed to the article and approved the submitted version.

## Supplementary material

Supplementary Table 1 Detailed statistical information for all experiments described.

Supplementary Figure 1 Blocking mAChRs does not affect excitatory synaptic transmission in SST interneurons. **(A)** Representative traces of sEPSCs recorded from an SST interneuron before and after the application of scopolamine (100 nM). **(B)** A histogram shows the effect of scopolamine on sEPSC frequency in the SST interneuron. **(C,D)** Summarized data show the effect of scopolamine on sEPSC (*n* = 7 cells/7 mice) and mEPSC (*n* = 7 cells/4 mice) frequencies and amplitudes, respectively. **(E)** An experiment shows the effect of scopolamine on evoked EPSCs. Inserts: the traces of evoked EPSCs taken at the times marked with a and b in the graph. **(F,G)** Summarized data show the effect of scopolamine on evoked EPSC (*n* = 11 cells/6 mice) amplitude and paired-pulse ratio, respectively.

Supplementary Figure 2 Blocking mAChRs does not affect excitatory synaptic transmission in PV interneurons. **(A)** Representative traces of sEPSCs recorded from a PV interneuron before and 10 min after application of scopolamine. **(B)** A histogram shows the effect of scopolamine on sEPSC frequency in the PV interneurons. **(C,D)** Summarized data show the effect of scopolamine on sEPSC (*n* = 7 cells/6 mice) and mEPSC (*n* = 6 cells/5 mice) frequencies and amplitudes, respectively. **(E)** An experiment shows the effect of scopolamine on evoked EPSCs. Inserts: the traces of evoked EPSCs taken at the time indicated by a and b in the graph. **(F,G)** Summarized data show the effect of scopolamine on evoked EPSC (*n* = 8 cells/6 mice) amplitude and paired-pulse ratio, respectively.

Supplementary Figure 3 Effects of Mus and Sco on frequency and amplitude of EPSCs and IPSCs. **(A,B)** Cumulative fraction of sEPSC inter-event interval and amplitude in SST and PV interneurons. **(C,D)** Cumulative fraction of mIPSC inter-event interval and amplitude in SST and PV interneurons.
