# Supplementary figures and images for: Cell-type-specific synaptic modulation of mAChR on SST and PV interneurons
Source: Front Psychiatry. 2023 Jan 12;13:1070478. doi: 10.3389/fpsyt.2022.1070478 (PMC9877455; doi:10.3389/fpsyt.2022.1070478)

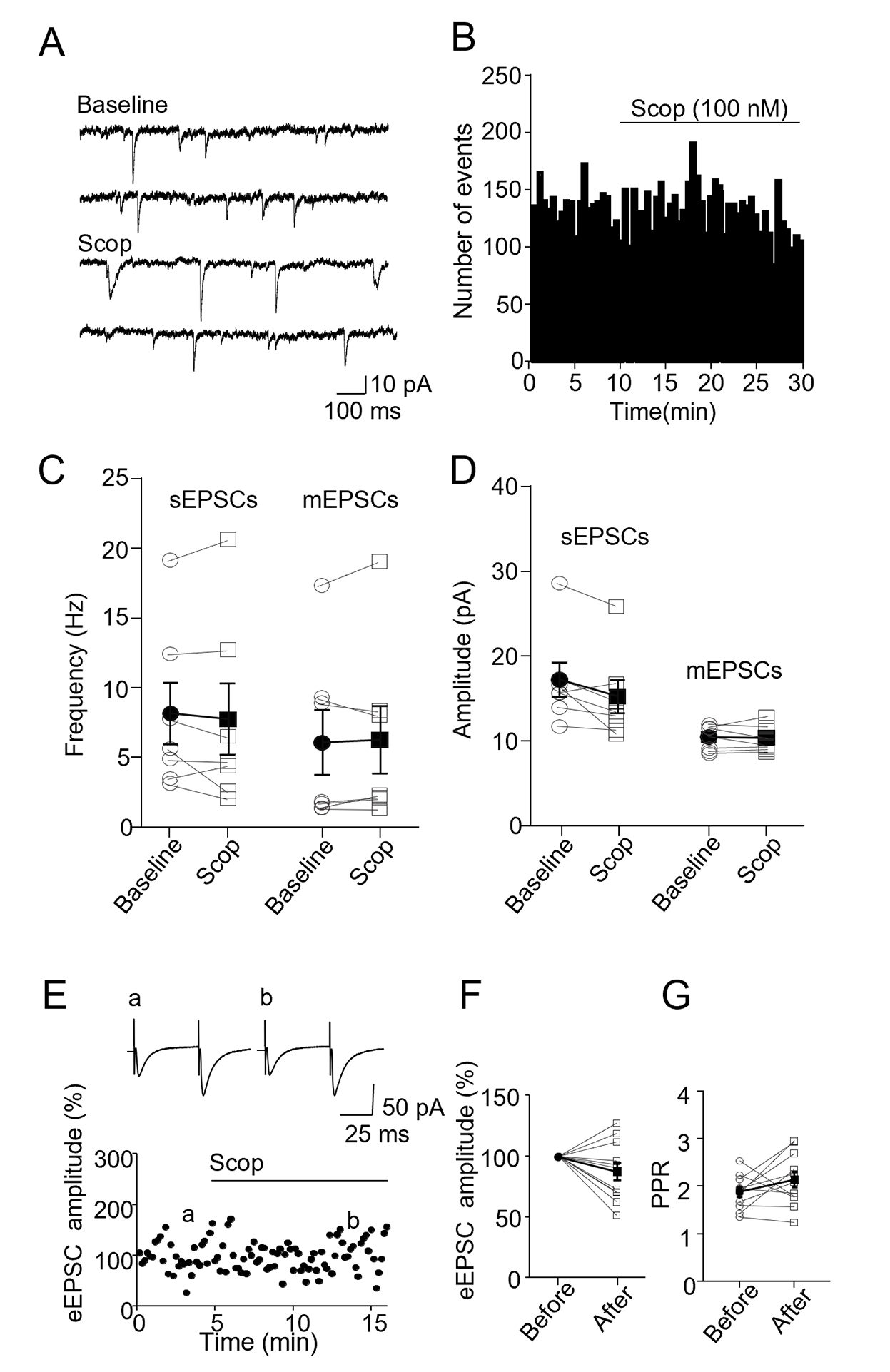

Supplement: Supplementary Figure 1 — Blocking mAChRs does not affect excitatory synaptic transmission in SST interneurons. (A) Representative traces of sEPSCs recorded from an SST interneuron before and after the application of scopolamine (100 nM). (B) A histogram shows the effect of scopolamine on sEPSC frequency in the SST interneuron. (C,D) Summarized data show the effect of scopolamine on sEPSC (n = 7 cells/7 mice) and mEPSC (n = 7 cells/4 mice) frequencies and amplitudes, respectively. (E) An experiment shows the effect of scopolamine on evoked EPSCs. Inserts: the traces of evoked EPSCs taken at the times marked with a and b in the graph. (F,G) Summarized data show the effect of scopolamine on evoked EPSC (n = 11 cells/6 mice) amplitude and paired-pulse ratio, respectively. [file Image_1.tif]

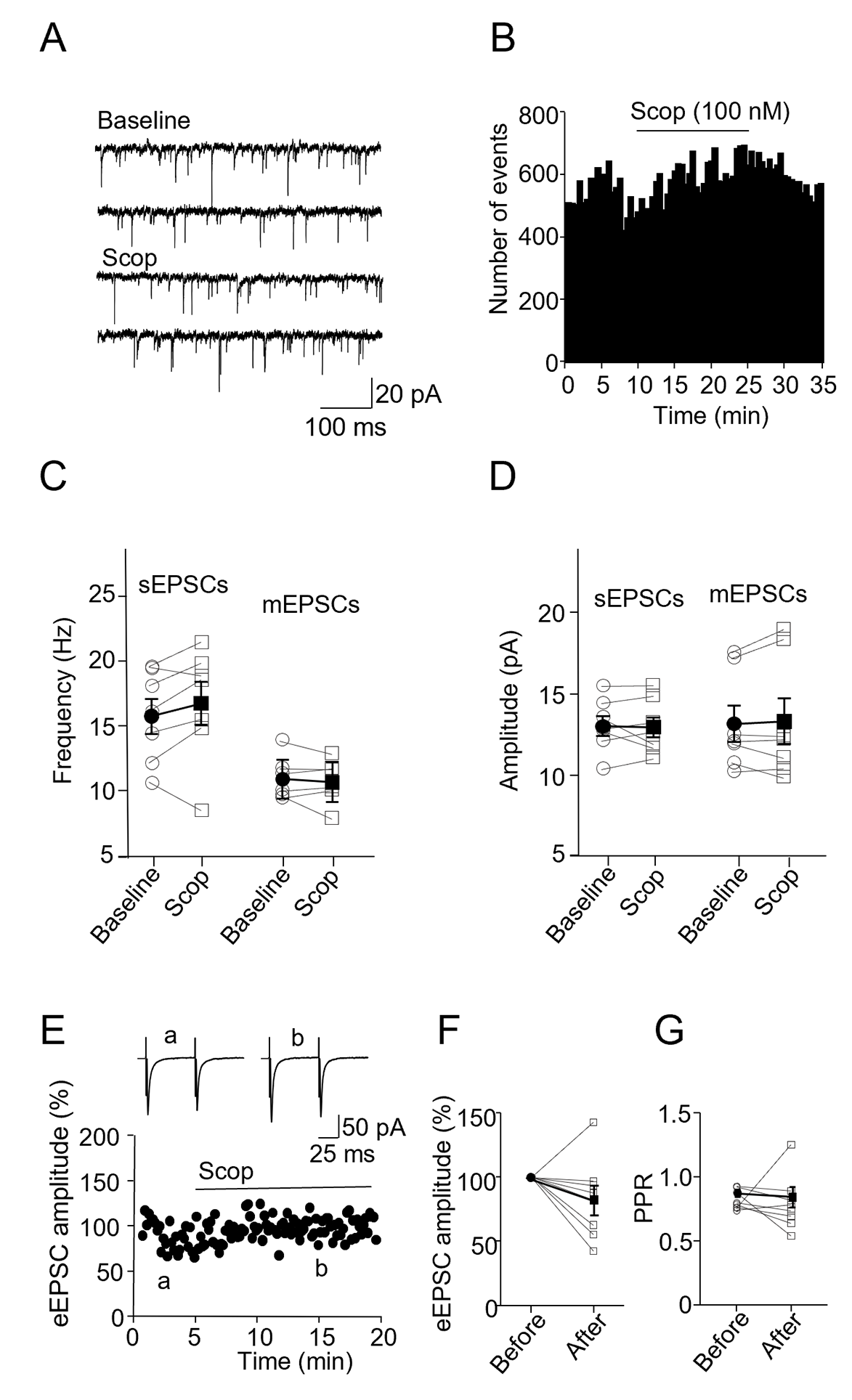

Supplement: Supplementary Figure 2 — Blocking mAChRs does not affect excitatory synaptic transmission in PV interneurons. (A) Representative traces of sEPSCs recorded from a PV interneuron before and 10 min after application of scopolamine. (B) A histogram shows the effect of scopolamine on sEPSC frequency in the PV interneurons. (C,D) Summarized data show the effect of scopolamine on sEPSC (n = 7 cells/6 mice) and mEPSC (n = 6 cells/5 mice) frequencies and amplitudes, respectively. (E) An experiment shows the effect of scopolamine on evoked EPSCs. Inserts: the traces of evoked EPSCs taken at the time indicated by a and b in the graph. (F,G) Summarized data show the effect of scopolamine on evoked EPSC (n = 8 cells/6 mice) amplitude and paired-pulse ratio, respectively. [file Image_2.tif]

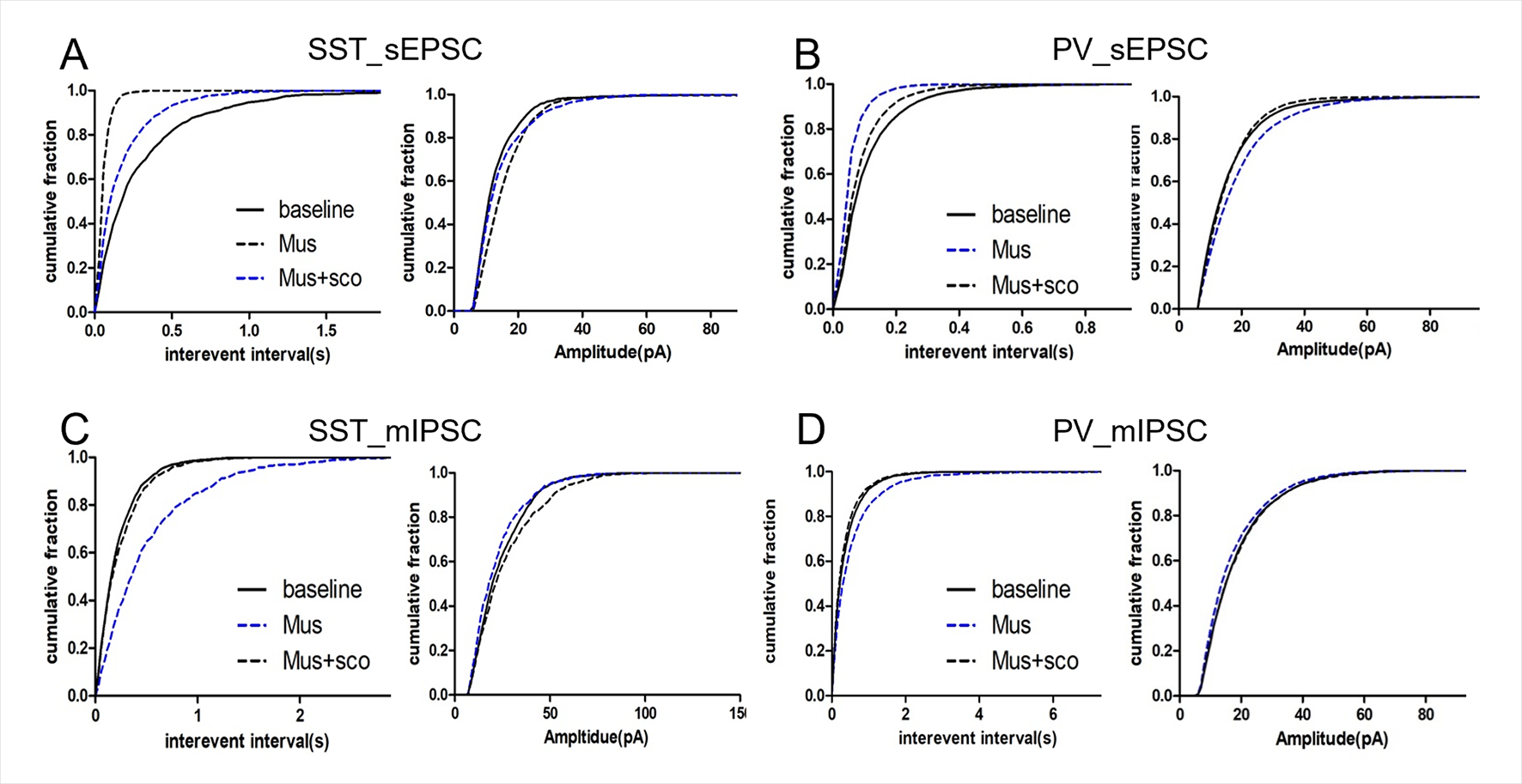

Supplement: Supplementary Figure 3 — Effects of Mus and Sco on frequency and amplitude of EPSCs and IPSCs. (A,B) Cumulative fraction of sEPSC inter-event interval and amplitude in SST and PV interneurons. (C,D) Cumulative fraction of mIPSC inter-event interval and amplitude in SST and PV interneurons. [file Image_3.tif]
